# Editorial: Continued opportunities in wearable technologies and physiological assessment

**DOI:** 10.3389/fdgth.2023.1166088

**Published:** 2023-03-28

**Authors:** James W. Navalta, Jennifer A. Bunn

**Affiliations:** ^1^ Department of Kinesiology and Nutrition Sciences, University of Nevada, Las Vegas, NV, United States; ^2^ College of Health Sciences, Sam Houston State University, Huntsville, TX, United States

**Keywords:** wearable technologies, physiology, accuracy and reliability, telehealth and clinical trials, free-living conditions

**Editorial on the Research Topic** Continued opportunities in wearable technologies and physiological assessment

We admit to facing a moment of vulnerability and not feeling confident of receiving any submissions to our Research Topic on Wearable Technologies and Physiological Assessment for the Frontiers in Digital Health journal when the call was announced in 2021. While wearable technology has been listed, since 2016, among the top trends (1) in our narrow field of interest (2, 3), we were hesitant about its broader appeal. Perhaps for those reasons we deliberately asked for a wide range of applications in which wearable technology could obtain physiological data, from telehealth and clinical trials to diversity, inclusion, and environmental health.

To our satisfaction, this Research Topic was honored with the work from outstanding research groups from around the globe, from Austria to Canada and from Australia to Italy. Boyer et al. reported on the ability of an axillary thermometer to provide temperature measurements, Tindale et al. detailed motivation and barriers for use of sensors in the workplace, Trost et al. evaluated the ability of wrist-worn devices to accurately determine movement intensity in children and adolescents, and Moscato et al. characterized sources of variability on photoplethysmographic (PPG) signals.

On the surface, the articles seem disparate and as varied as our initial call. In hindsight, this should not be surprising. It did present us with a brief moment of panic when we were invited to write an editorial under a single unifying theme. We read and re-read the articles. We walked away and reflected. We distracted ourselves with other work, and in a moment of clarity were provided with an epiphany.

The manuscripts presented in this Research Topic are united in difficulties that every one of us who performs work in the wearable technology and physiological assessment space is intimately familiar with—namely, limitations. The framing of limitations in the scientific literature is generally buried deep in discussion sections where the hope is that potential readers lose interest before they arrive at our list of publicly acknowledged flaws and shortcomings. For this editorial, we would like to present limitations as something else—opportunities.

Boyer et al. found the SteadyTemp® axillary thermometer to be susceptible to environmental disturbances and concluded that the device did not return accurate temperature measurements in their clinical population. Despite this outcome, the authors noted the opportunity to use the device in appropriate use-case scenarios. Another opportunity was exercised to reflect meaningfully on the arbitrary thresholds defining fever, and open a discussion into potential updates.

Tindale et al. reported that few individuals use wearable devices to monitor employees in the workplace, with 5% making use of body sensors and 1% using brain sensors. To those of us who conduct research in the wearable space, these were shockingly low percentages. While wearables may provide information regarding employee wellness and safety, there is an opportunity to address, prior to implementation, concerns surrounding fears about data privacy and how the information will be used.

Trost et al. discovered that while the ActiGraph GT3X+ could accurately determine when an adolescent participant was engaged in a sedentary pursuit, the device fell short when activity was performed (light, moderate, as well as vigorous physical activity). These findings highlight an opportunity for the manufacturers of wearable technology devices to invest time and effort into the accuracy of their products in a wide array of use cases. We understand the temptation, based on financial incentives, that companies face to distribute products into circulation as quickly as possible. Because wearable devices are used by individuals most often outside of a sterile laboratory condition, particular time and emphasis should be paid to ensuring that devices are accurate in free-living conditions.

Moscato et al. provided evidence that several factors affect the PPG signaling of the Empatica E4, most importantly, physical activity and health status. The findings have wide-reaching consequences, as many heart rate-based devices rely on PPG technology to return measurements. Because of this, there is an opportunity for the industry to collaborate with scientists to refine and extend the capabilities of current technologies, and to develop devices with new technology.

As this is an editorial, we will take the liberty to propose many other opportunities. There is an opportunity for journals and reviewers of manuscripts presenting findings on physiological variables obtained from wearable devices to reframe their mindset about what constitutes acceptable results. Not all devices will meet a predetermined threshold for accuracy. This may not mean the study design was flawed or conducted in an inappropriate manner. Despite no “significant results” to report, the information should be disseminated, if for no other reason than to spare future researchers the time investment of needlessly replicating the study. Additionally, authors should not feel the need to resort to or be asked by reviewers to present their wearable data as an adjunct to what is perceived as more meaningful findings.

We state in no uncertain terms that research with wearables providing physiological data is important. There is an opportunity for institutional administrators to understand the importance of applied research, whether they perceive the direction to be fundable or not. There is an opportunity for researchers to continue to communicate the importance of the work being conducted. Toward this end, there is an associated opportunity for investigators and researchers to train the next generation of students and future collaborators in the skills necessary to continue conducting this type of work.

Finally, there is an opportunity for an open discussion on the limitations that are inherent in studies incorporating physiological measurements through wearable devices, and the best way to acknowledge them. Being fully transparent will prevent others from repeating avoidable pitfalls and allow the field to conduct the high-quality research that is needed.
